# The FGF23–Undercarboxylated Osteocalcin Axis in Predialysis Chronic Kidney Disease: Linking Mineral Homeostasis and Bone Turnover

**DOI:** 10.3390/ijms27219743

**Published:** 2026-10-30

**Authors:** Tatyana Simeonova, Borislav Ignatov, Tsvetelina Eftimova, Alexander Blazhev, Krasimir Kostov

**Affiliations:** 1 Department of Physiology and Pathophysiology, Medical University–Pleven, 1 Kliment Ohridski Str., 5800 Pleven, Bulgaria; dr_tsimeonova@abv.bg (T.S.); dr_bignatov@abv.bg (B.I.); 2 Hemodialysis Unit, St. Marina Hospital, 5800 Pleven, Bulgaria; dr_eftimova@abv.bg; 3 Department of Biomedical Science, Faculty of Veterinary Medicine, Medical University–Pleven, 1 Kliment Ohridski Str., 5800 Pleven, Bulgaria; alexander.blazhev@mu-pleven.bg

**Keywords:** FGF23, undercarboxylated osteocalcin, chronic kidney disease, CKD-MBD, bone–kidney axis, bone turnover

## Abstract

Fibroblast growth factor 23 (FGF23) and osteocalcin are involved in mineral and bone metabolism, but their relationship in chronic kidney disease (CKD) remains insufficiently characterized. This cross-sectional study investigated the association between FGF23 and undercarboxylated osteocalcin (Glu-OC) and whether this association persisted after accounting for kidney function. The study included 80 adults: 12 healthy controls and 68 patients with non-dialysis CKD stages G1–G4. Associations were assessed using Spearman correlation, partial correlation adjusted for estimated glomerular filtration rate (eGFR), and complementary multivariable linear regression models. Among patients with CKD, FGF23 was inversely correlated with eGFR (ρ = −0.346, *p* = 0.004) and Glu-OC (ρ = −0.344, *p* = 0.004), and positively correlated with serum creatinine (ρ = 0.299, *p* = 0.013). The inverse association between FGF23 and Glu-OC persisted after adjustment for eGFR (partial ρ = −0.475, *p* < 0.001). In the multivariable regression models, Glu-OC was inversely associated with FGF23 (standardized β = −0.258, *p* = 0.024), whereas alkaline phosphatase was positively associated with FGF23 (β = 0.369, *p* = 0.001). In the model with Glu-OC as the dependent variable, FGF23 (β = −0.266, *p* = 0.020) and serum calcium (β = −0.366, *p* = 0.002) were inversely associated with Glu-OC. Significant inverse correlations between FGF23 and Glu-OC were observed in CKD stages G1–G3, but not in stage G4 or the control group. These findings support an association between FGF23 and Glu-OC in non-dialysis CKD and provide a rationale for further investigation of a potential FGF23–Glu-OC axis in CKD-associated mineral and bone disorder (CKD-MBD). Longitudinal and mechanistic studies are needed to clarify the nature and clinical relevance of this association.

## 1. Introduction

Chronic kidney disease–mineral and bone disorder (CKD-MBD) is a systemic syndrome characterized by disturbances in mineral metabolism, abnormalities in bone turnover, mineralization and structure, as well as vascular calcification [1,2]. Early disturbances reflect alterations in kidney–bone interactions and may progress alongside phosphate retention, changes in fibroblast growth factor 23 (FGF23) signaling, impaired vitamin D metabolism, and the development of secondary hyperparathyroidism [3,4,5].

Osteocalcin is a non-collagenous protein synthesized by osteoblast-lineage cells and is associated with bone formation. Vitamin K-dependent γ-carboxylation produces carboxylated osteocalcin (Gla-OC), whereas partially or fully undercarboxylated forms circulate as undercarboxylated osteocalcin (Glu-OC) [6,7]. Specifically, Gla-OC is characterized by a fixed number of three carboxylations, whereas the number of carboxylations in Glu-OC is variable, ranging from zero to two. Osteocalcin may participate in endocrine communication [8,9], and Glu-OC has been investigated in relation to glucose metabolism and insulin sensitivity [9,10]. However, findings from human studies have been less consistent than those reported in experimental models [8,11,12]. FGF23 is a bone-derived endocrine hormone produced by osteocytes and osteoblast-lineage cells that acts through fibroblast growth factor receptors in the presence of α-Klotho. It promotes renal phosphate excretion and suppresses renal synthesis of 1,25-dihydroxyvitamin D [13,14,15]. FGF23 concentrations increase as kidney function declines, a change generally considered an early adaptive response to disturbances in phosphate homeostasis [5,14,16]. Elevated FGF23 levels have been associated with adverse clinical outcomes, although the relative contributions of direct FGF23 effects and concomitant pathophysiological changes in CKD-MBD remain unclear [5,14,17,18].

FGF23 and osteocalcin are linked to the biology of osteoblast–osteocyte lineage cells, providing a biological rationale for investigating a potential relationship between them [19,20]. Experimental studies suggest that FGF23 signaling may influence osteoblast-lineage cell activity and bone remodeling [21], while FGF23 production by osteoblasts and osteocytes is regulated by various mineral and hormonal signals [15,22,23]. In CKD, disturbances in phosphate and vitamin D homeostasis coexist with alterations in bone turnover that may potentially affect both FGF23 concentrations and circulating Glu-OC levels. However, this biological plausibility does not establish a direct regulatory relationship between the two biomarkers, as any observed association may also reflect shared changes in mineral metabolism and osteoblast-lineage cell activity.

The potential clinical relevance of Glu-OC extends beyond bone metabolism. An observational study in patients with non-dialysis CKD reported an association between lower circulating undercarboxylated osteocalcin levels and carotid atherosclerosis [24]. This finding does not establish a causal role for Glu-OC in vascular disease, nor does it demonstrate a shared regulatory mechanism between FGF23 and Glu-OC. Nevertheless, it supports clinical interest in investigating these biomarkers in the context of the interplay between bone metabolism and cardiovascular abnormalities in CKD.

Despite increasing knowledge of the roles of FGF23 and osteocalcin in CKD, the relationship between circulating FGF23 and Glu-OC, particularly after accounting for kidney function, remains insufficiently characterized [25,26]. Reduced kidney function may influence circulating FGF23 concentrations through changes in renal clearance and other disease-related processes [27,28]. Consequently, the observed association between FGF23 and Glu-OC may partly reflect differences in kidney function or CKD severity rather than a specific relationship between the two biomarkers [18,26]. Therefore, assessing this association after adjustment for estimated glomerular filtration rate (eGFR), as well as investigating potential differences across CKD stages, is important for the appropriate interpretation of the findings.

As CKD progresses, phosphate retention, impaired vitamin D metabolism, changes in parathyroid regulation, and alterations in serum calcium concentrations may contribute to changes in bone turnover [29,30,31]. Calcium is an essential component of bone mineralization, and osteocalcin participates in bone matrix biology [32]. It remains unclear whether serum calcium is independently associated with circulating Glu-OC and whether serum calcium and bone turnover markers contribute to a more comprehensive characterization of the association between FGF23 and Glu-OC. Investigating these relationships may help clarify the mineral and bone metabolic context in which this association occurs.

Based on these considerations, we hypothesized that circulating FGF23 is associated with Glu-OC in adults with non-dialysis CKD and that this association may persist after adjustment for kidney function. The primary aim of the study was to investigate this association and assess its independence from eGFR. The secondary aims were to examine the associations of FGF23 and Glu-OC with serum calcium and bone turnover markers and to characterize these relationships across CKD stages. The hypothesis concerns a statistical association between circulating biomarkers and does not presuppose a direct causal relationship or the existence of a feedback mechanism.

## 2. Results

### 2.1. Clinical and Biochemical Characteristics of the Study Population

Comparison of the measured parameters across groups stratified by the degree of kidney dysfunction revealed no statistically significant difference in sex distribution (*p* = 0.152), whereas age differed significantly between groups (*p* < 0.001). As expected, eGFR progressively decreased from 103.00 mL/min/1.73 m^2^ in controls to 16.50 mL/min/1.73 m^2^ in CKD G4, accompanied by a significant increase in serum creatinine (*p* < 0.001).

Significant changes were observed in mineral metabolism parameters. Serum calcium decreased, whereas inorganic phosphate and PTH increased with worsening kidney dysfunction (*p* = 0.004, *p* < 0.001, and *p* < 0.001, respectively). These changes were most pronounced in CKD G4, where median Ca, Pi, and PTH values were 2.31 mmol/L, 1.52 mmol/L, and 110.35 pg/mL, respectively. Total 25(OH)D concentrations also decreased significantly across groups (*p* = 0.001), while ALP tended to increase with more advanced kidney dysfunction (*p* = 0.012).

Glu-OC showed statistically significant between-group differences (*p* = 0.033), increasing from 0.59 ng/mL in controls to 5.22 ng/mL in CKD G3, followed by a slight decrease in CKD G4. Gla-OC also differed significantly between groups (*p* = 0.035), but without a clear progressive trend. FGF23 showed significant between-group differences (*p* = 0.023), with the lowest median in CKD G1 (83.40 pg/mL) and the highest in CKD G4 (208.65 pg/mL), suggesting a nonlinear pattern with CKD progression. In contrast, α-Klotho did not differ significantly between groups (*p* = 0.722). P1NP increased significantly with worsening kidney dysfunction (*p* < 0.001), reaching 5450 pg/mL in CKD G4. CTX1 also showed statistically significant between-group differences (*p* = 0.033), but without a consistent trend.

In summary, CKD progression was associated with characteristic disturbances in mineral and bone metabolism, including decreases in calcium and total 25(OH)D, increases in phosphate, PTH, and ALP, and a significant increase in P1NP. Significant between-group differences in Glu-OC, Gla-OC, and FGF23 further support the presence of altered regulation of bone and mineral metabolism with worsening kidney dysfunction. The clinical and biochemical characteristics of the study population are summarized in Table 1.

The distribution of CKD etiologies varied across disease stages. Hypertensive and diabetic nephropathies were the most frequently identified etiologies, accounting for 24 (35.3%) and 23 (33.8%) of the 68 patients with CKD, respectively. Chronic glomerulonephritis was identified in 9 patients (13.2%), chronic interstitial nephritis in 5 (7.4%), chronic pyelonephritis in 4 (5.9%), and polycystic kidney disease in 3 (4.4%).

To assess the individual dynamics of FGF23 and Glu-OC across the different groups, a post-hoc analysis was performed using the Dunn–Bonferroni test (Table 2).

Despite the apparent differences in both parameters between the control group and stage G1 (an approximately fivefold increase in Glu-OC and a twofold decrease in FGF23), no statistically significant differences were identified between these groups after adjustment for multiple comparisons. For FGF23, a statistically significant difference was observed only between groups G1 and G4 (adjusted *p* = 0.036). For Glu-OC, a difference was observed between the control group and G4, reaching the borderline threshold of statistical significance (adjusted *p* = 0.050). The remaining pairwise comparisons did not reveal statistically significant differences after Bonferroni correction. These findings suggest that the observed differences in FGF23 and Glu-OC levels were not statistically significant across most group comparisons, with the most pronounced differences observed between the early and advanced stages of chronic kidney disease for FGF23 and between healthy controls and stage G4 for Glu-OC.

### 2.2. Associations of FGF23 and Glu-OC with Renal Function and Mineral-Bone Metabolism

**Figure 1:**
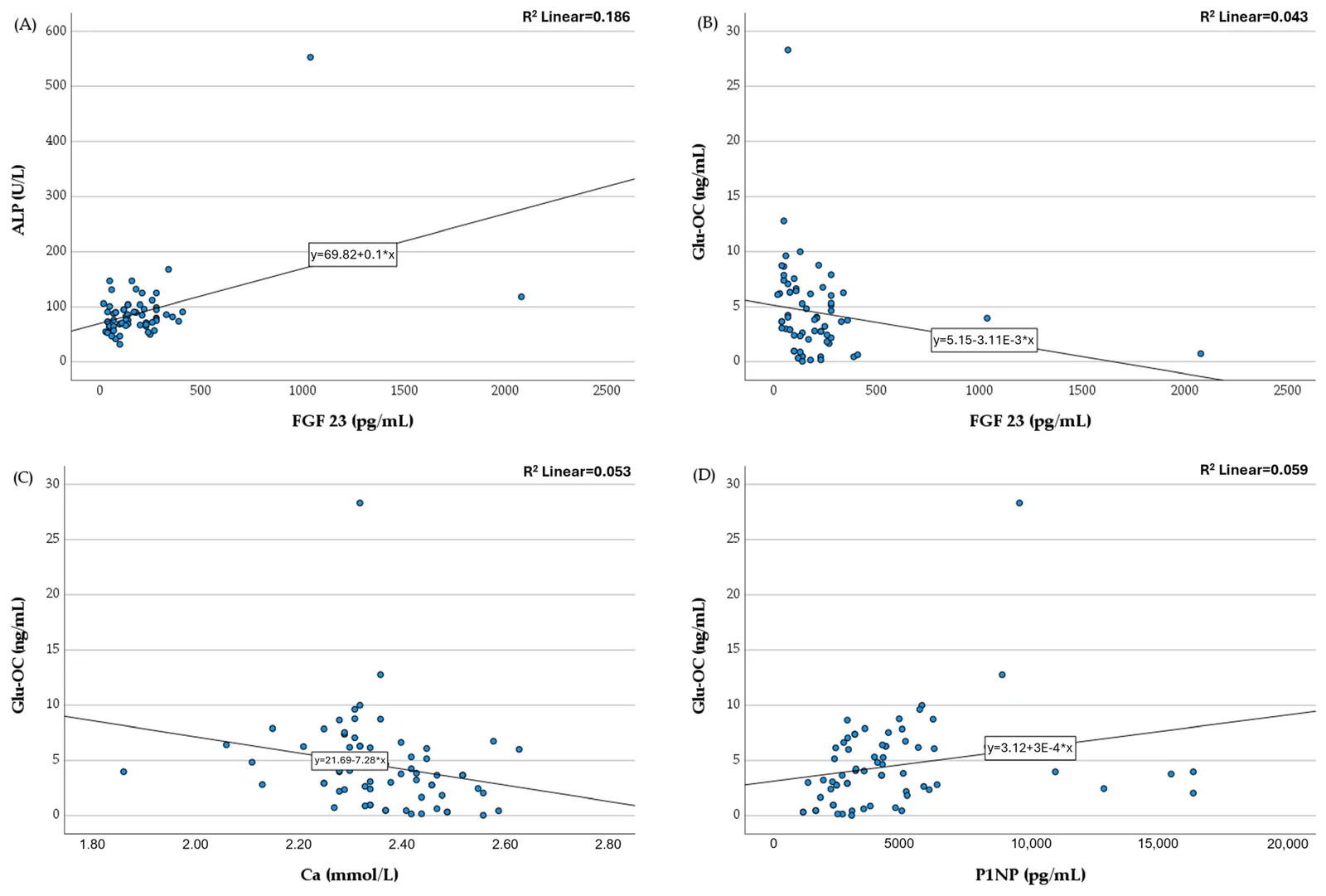
Associations between serum biomarkers in patients with chronic kidney disease (CKD; *n* = 68). Scatter plots show the relationships between (**A**) alkaline phosphatase (ALP) and fibroblast growth factor 23 (FGF23), (**B**) undercarboxylated osteocalcin (Glu-OC) and FGF23, (**C**) Glu-OC and total serum calcium, and (**D**) Glu-OC and procollagen type I N-terminal propeptide (P1NP). The corresponding coefficients of determination (R^2^) were 0.186, 0.043, 0.053, and 0.059, respectively.

Correlation analysis revealed significant associations of FGF23 and Glu-OC with kidney function and selected parameters of mineral metabolism and bone turnover in patients with CKD (*n* = 68) (Table 3 and Figure 1). FGF23 was negatively correlated with eGFR (ρ = −0.346, *p* = 0.004) and positively correlated with serum creatinine (ρ = 0.299, *p* = 0.013), indicating that higher FGF23 concentrations were associated with poorer kidney function. FGF23 was also inversely correlated with Glu-OC (ρ = −0.344, *p* = 0.004) and positively correlated with serum phosphate (ρ = 0.257, *p* = 0.034) and total alkaline phosphatase (ALP; ρ = 0.286, *p* = 0.018). No statistically significant unadjusted associations were observed between FGF23 and P1NP, PTH, serum calcium, total 25(OH)D, α-Klotho, or CTX-1.

After adjustment for eGFR using rank-transformed variables, the inverse association between FGF23 and Glu-OC remained statistically significant (partial correlation coefficient = −0.475, *p* < 0.001). FGF23 was also inversely associated with P1NP (partial correlation coefficient = −0.284, *p* = 0.020) and positively associated with serum calcium (partial correlation coefficient = 0.271, *p* = 0.027). The association between FGF23 and ALP was attenuated and did not reach statistical significance after adjustment (partial correlation coefficient = 0.232, *p* = 0.059). No statistically significant adjusted associations were observed between FGF23 and PTH, serum phosphate, total 25(OH)D, α-Klotho, or CTX-1.

Glu-OC was negatively correlated with eGFR (ρ = −0.252, *p* = 0.038) and positively correlated with serum creatinine (ρ = 0.272, *p* = 0.025). Among the investigated markers of bone turnover and mineral metabolism, Glu-OC was positively correlated with P1NP (ρ = 0.382, *p* = 0.001) and negatively correlated with serum calcium (ρ = −0.405, *p* < 0.001). No statistically significant unadjusted associations were observed between Glu-OC and PTH, serum phosphate, total 25(OH)D, α-Klotho, CTX-1, or ALP.

After adjustment for eGFR, the positive association between Glu-OC and P1NP remained statistically significant (partial correlation coefficient = 0.297, *p* = 0.015), as did the inverse association between Glu-OC and serum calcium (partial correlation coefficient = −0.382, *p* = 0.001). The inverse association between Glu-OC and FGF23 also persisted (partial correlation coefficient = −0.475, *p* < 0.001). No statistically significant adjusted associations were observed between Glu-OC and PTH, serum phosphate, total 25(OH)D, α-Klotho, CTX-1, or ALP.

Overall, the findings demonstrate an inverse association between FGF23 and Glu-OC that persisted after adjustment for eGFR. The adjusted analyses also identified an inverse association between FGF23 and P1NP and a positive association between FGF23 and serum calcium, whereas Glu-OC remained positively associated with P1NP and inversely associated with serum calcium. These findings indicate that the observed associations with selected markers of bone turnover and mineral metabolism persisted after statistical adjustment for eGFR. Nevertheless, given the cross-sectional design, the results do not establish causal relationships or identify the underlying biological mechanisms.

Given the absence of statistically significant correlations involving circulating α-Klotho, an additional sensitivity analysis was performed to examine whether adjustment for α-Klotho altered the inverse association between FGF23 and Glu-OC. Partial correlation models were constructed with adjustment for eGFR alone, eGFR and α-Klotho jointly, and α-Klotho alone. This approach allowed assessment of the robustness of the observed association under alternative adjustment models, while recognizing that circulating α-Klotho concentrations may not fully reflect tissue-specific Klotho expression or activity. The results are presented in Table 4.

The inverse association between FGF23 and Glu-OC remained statistically significant after adjustment for eGFR (partial correlation coefficient = −0.475, *p* < 0.001) and was similar after additional adjustment for α-Klotho (partial correlation coefficient = −0.481, *p* < 0.001). When adjustment was limited to α-Klotho, the association remained statistically significant but was weaker (partial correlation coefficient = −0.346, *p* = 0.004). These findings are consistent with the persistence of the inverse association across the examined adjustment models, although the differences between correlation coefficients were not formally tested.

### 2.3. Association Between FGF23 and Glu-OC in Controls and Across CKD Stages

Spearman correlation analyses were performed to assess the association between fibroblast growth factor 23 (FGF23) and undercarboxylated osteocalcin (Glu-OC) in the control group and in patients with chronic kidney disease (CKD) stages G1–G4. Significant inverse correlations were observed in CKD G1 (ρ = −0.768, *p* < 0.001), G2 (ρ = −0.511, *p* = 0.043), and G3 (ρ = −0.541, *p* = 0.021). In CKD G4, the correlation was also inverse but did not reach statistical significance (ρ = −0.366, *p* = 0.163). No statistically significant correlation was observed in the control group (ρ = −0.086, *p* = 0.791). The correlation coefficients, corresponding *p*-values, and post-hoc power estimates are presented in Table 5.

The post-hoc power estimates varied across groups, with the highest estimate observed in CKD G1 and lower estimates in CKD G2–G4 and the control group. These estimates should be interpreted cautiously because post-hoc power depends on the observed effect size and does not independently establish the reliability of the findings. Accordingly, the absence of statistical significance in CKD G4 and the control group should not be interpreted as definitive evidence that an association is absent.

To further characterize the uncertainty surrounding the group-specific correlation coefficients, a bootstrap analysis based on 10,000 resamples was performed. The bias-corrected and accelerated (BCa) 95% confidence intervals excluded zero in CKD G1, G2, and G3, whereas the intervals for CKD G4 and the control group included zero. The upper confidence limit for CKD G2 was close to zero (−0.002). Bootstrap standard errors and corresponding confidence intervals are presented in Table 6.

Overall, statistically significant inverse associations between FGF23 and Glu-OC were observed in CKD stages G1–G3, whereas the associations in CKD G4 and the control group did not reach statistical significance. These findings describe the associations observed within each group; however, differences in statistical significance across groups do not establish that the correlation coefficients differ significantly between CKD stages. Direct statistical comparisons between correlation coefficients would be required to assess such differences.

### 2.4. Multivariable Linear Regression Analysis of the Reciprocal Association Between FGF23 and Glu-OC

To further investigate the relationship between FGF23 and Glu-OC while accounting for potential clinical and biochemical covariates, multivariable linear regression analysis with backward elimination was performed, with serum FGF23 concentration as the dependent variable (Table 7). The final model retained eGFR, ALP, and Glu-OC. The model was statistically significant (F(3,64) = 7.842; *p* < 0.001) and explained 26.9% of the variance in FGF23 concentrations (R^2^ = 0.269; adjusted R^2^ = 0.235).

ALP showed a significant positive association with FGF23 (β = 0.369; *p* = 0.001), whereas Glu-OC was significantly and negatively associated with FGF23 concentration (β = −0.258; *p* = 0.024). eGFR showed a negative association with FGF23 that approached statistical significance (β = −0.226; *p* = 0.053).

Thus, after adjustment for the other predictors in the final model, Glu-OC remained independently and negatively associated with FGF23, while ALP showed an independent positive association with FGF23. The association between eGFR and FGF23 was negative and approached statistical significance.

To assess the association in the reverse direction, a second multivariable linear regression model with backward elimination was fitted, with Glu-OC concentration as the dependent variable (Table 8). The final model was statistically significant (F(3,64) = 6.409; *p* = 0.001) and explained 23.1% of the variance in Glu-OC concentrations (R^2^ = 0.231; adjusted R^2^ = 0.195). Age showed a significant positive association with Glu-OC (β = 0.353; *p* = 0.003), whereas serum calcium (β = −0.366; *p* = 0.002) and FGF23 (β = −0.266; *p* = 0.020) were independently and negatively associated with Glu-OC concentrations.

In both complementary multivariable models, the negative association between FGF23 and Glu-OC persisted after adjustment for the other clinical and biochemical parameters examined. In the model with FGF23 as the dependent variable, Glu-OC was independently and negatively associated with FGF23 concentration (β = −0.258; *p* = 0.024), while ALP showed a significant positive association with FGF23 (β = 0.369; *p* = 0.001), and eGFR showed a negative association that approached statistical significance (β = −0.226; *p* = 0.053). In the reverse model, with Glu-OC as the dependent variable, FGF23 was also independently and negatively associated with Glu-OC concentrations (β = −0.266; *p* = 0.020), while serum calcium showed a significant negative association (β = −0.366; *p* = 0.002), and age showed a significant positive association (β = 0.353; *p* = 0.003).

## 3. Discussion

### 3.1. FGF23, Renal Function and Bone Turnover

The present study demonstrates that serum FGF23 is associated with kidney function and markers of bone metabolism in patients with non-dialysis chronic kidney disease (CKD). FGF23 correlated negatively with eGFR and positively with serum creatinine (Table 3), consistent with the association between declining kidney function and alterations in FGF23 regulation [33]. Although the increase in FGF23 across CKD stages was not linear (Table 1), its overall association with kidney function supports its role as a component of the adaptive response to disturbances in mineral homeostasis in CKD [33,34].

Of particular interest is the positive association between FGF23 and ALP, which remained statistically significant after accounting for the other factors examined in the multivariable model. ALP showed the strongest standardized association with FGF23 (β = 0.369, *p* = 0.001; Table 7 and Figure 1). This finding supports the concept that FGF23 should be viewed not solely as a regulator of phosphate metabolism but also as part of a broader functional relationship linking kidney function, bone turnover, and mineral regulation [34,35]. An association between circulating FGF23 concentrations and histomorphometric indices of bone turnover and mineralization has also been reported in a bone biopsy study involving patients receiving dialysis [35], although these findings are not directly equivalent to those in non-dialysis CKD.

The interpretation of this association requires distinguishing between FGF23 as a potential contributor to bone remodeling abnormalities and FGF23 as a circulating marker of the underlying CKD-MBD phenotype. FGF23 is produced predominantly by osteocytes and osteoblast-lineage cells and functions as a central regulator of phosphate and vitamin D homeostasis; however, its production is also influenced by systemic mineral and hormonal signals [15,33]. Experimental evidence further suggests that FGF23 may affect osteoblast-lineage cell activity through inhibition of Wnt signaling, potentially contributing to impaired bone formation in CKD [21]. Nevertheless, these experimental findings do not establish that circulating FGF23 directly causes high-turnover bone disease in humans.

In the present study, the association between FGF23 and ALP after adjustment for the other variables included in the multivariable model is compatible with a link between FGF23 and bone-related metabolic activity, but ALP alone cannot establish the presence or type of renal osteodystrophy. Because the study did not include bone histomorphometry or a bone-specific ALP measurement, the observed association cannot distinguish a direct contribution of FGF23 to skeletal abnormalities from a secondary response to the mineral, hormonal, and bone-turnover disturbances accompanying CKD-MBD.

Nevertheless, owing to the cross-sectional design, the present data cannot establish whether increased bone turnover contributes to higher FGF23 concentrations, whether FGF23 influences bone cell activity, or whether the observed association reflects the interplay between these processes.

### 3.2. Independent Inverse Association Between FGF23 and Glu-OC

The main finding of the present study is the inverse association between FGF23 and Glu-OC. In the CKD cohort, FGF23 correlated negatively with Glu-OC (ρ = −0.344, *p* = 0.004), and this association became stronger after adjustment for eGFR (partial correlation coefficient = −0.475, *p* < 0.001; Table 3 and Figure 1). In addition, Glu-OC remained inversely associated with FGF23 concentrations in the multivariable regression model with FGF23 as the dependent variable (β = −0.258, *p* = 0.024; Table 7). A consistent inverse association was also observed in the second regression model, with Glu-OC as the dependent variable (β = −0.266, *p* = 0.020; Table 8). Together, these complementary analyses support an inverse association between FGF23 and Glu-OC after accounting for the covariates included in the respective models.

The persistence of this association after adjustment for eGFR suggests that differences in kidney function alone do not fully account for the observed relationship in the present cohort. Furthermore, the inverse association remained significant after simultaneous adjustment for eGFR and circulating α-Klotho (partial correlation coefficient = −0.481, *p* < 0.001; Table 4), indicating that it was not fully explained by these measured variables. However, this finding does not establish biological independence from Klotho-dependent signaling. Given the cross-sectional design, the observed association does not demonstrate a direct biological interaction and cannot distinguish such an interaction from shared regulatory influences.

The observed relationship is biologically plausible because FGF23 is produced primarily by cells of the osteoblast/osteocyte lineage and participates in the regulation of mineral metabolism and bone cell function [34,35]. Experimental evidence also suggests that FGF23 signaling may influence the differentiation and functional state of osteoblast-lineage cells [34,36]. Nevertheless, the present human data do not directly demonstrate that FGF23 suppresses osteocalcin production or alters its carboxylation status. Accordingly, the findings support an inverse association between FGF23 and Glu-OC that may reflect changes in osteoblast/osteocyte lineage function, while the underlying mechanisms remain to be established.

### 3.3. Stage-Dependent Relationship Between FGF23 and Glu-OC

The stage-stratified analysis provides additional insight into the association between FGF23 and Glu-OC across CKD stages. Significant inverse correlations were observed in CKD G1 (ρ = −0.768, *p* < 0.001), G2 (ρ = −0.511, *p* = 0.043), and G3 (ρ = −0.541, *p* = 0.021) (Table 5). Although these correlations were statistically significant in G1–G3, differences in statistical significance across stage groups do not establish that the correlation coefficients differ significantly between stages.

These findings can be considered alongside the descriptive differences in Glu-OC and FGF23 concentrations observed in CKD G1 compared with controls. Glu-OC concentrations were higher, whereas FGF23 concentrations were lower in G1 (Table 1). However, neither difference between controls and G1 reached statistical significance in the post hoc comparisons after adjustment for multiple testing. The only statistically significant pairwise difference in FGF23 was observed between CKD G1 and G4 (Table 2). The approximately fivefold higher median Glu-OC concentration in G1 compared with controls (2.93 vs. 0.59 ng/mL), despite relatively preserved kidney function, may be compatible with early alterations in osteoblast activity or vitamin K-dependent osteocalcin carboxylation, although these mechanisms were not directly assessed in the present study. The lower median FGF23 concentration in G1 may reflect differences in phosphate regulation before the more pronounced disturbances that can accompany advanced CKD. However, preserved phosphate homeostasis cannot be inferred from these findings alone.

The inverse correlations between FGF23 and Glu-OC observed in CKD G1–G3 may reflect an association between these biomarkers in the context of CKD-related mineral and bone metabolic alterations, without necessarily indicating a direct causal relationship. In CKD G4, the correlation remained negative but did not reach statistical significance (ρ = −0.366, *p* = 0.163; Table 5). This finding should not automatically be interpreted as evidence that the association between FGF23 and Glu-OC is absent in advanced CKD. Several factors may contribute to the observed pattern, including more pronounced disturbances in calcium–phosphate homeostasis, changes in PTH and vitamin D, differences in bone turnover, and greater individual heterogeneity in the CKD-MBD phenotype [37]. Furthermore, the relatively small number of participants in each stage group limits the precision of the stage-specific correlation estimates, particularly in G4.

Overall, the findings support an inverse association between FGF23 and Glu-OC within CKD stages G1–G3, whereas the estimate for G4 remains statistically uncertain. Whether the strength or determinants of this association change as CKD progresses cannot be established from the present cross-sectional data. Larger studies with adequate representation across CKD stages, formal comparisons of correlation coefficients, and longitudinal measurements are needed to investigate whether the observed pattern reflects stage-related biological changes.

### 3.4. Glu-OC, Calcium, and Bone Formation

Another important finding was the inverse association between total serum calcium and Glu-OC. Glu-OC correlated negatively with calcium (ρ = −0.405, *p* < 0.001), and this relationship remained significant after adjustment for eGFR (partial ρ = −0.382, *p* = 0.001; Table 3 and Figure 1). In the multivariable model, calcium also remained an independent predictor of Glu-OC (β = −0.366, *p* = 0.002; Table 8).

This observation is of interest in the context of progressive CKD-MBD, in which declining eGFR is accompanied by changes in calcium–phosphate homeostasis and vitamin D–PTH regulation [37]. In the present cohort, lower calcium concentrations in more advanced CKD were accompanied by higher Glu-OC concentrations. However, this relationship should not be interpreted as evidence that serum calcium directly controls osteocalcin carboxylation.

The observed association may reflect a more complex interplay among mineral availability, bone mineralization, the interaction of osteocalcin with bone mineral, and osteoblast-lineage cell activity. Therefore, the specific mechanism linking calcium status to Glu-OC remains undetermined and requires investigation in future studies.

An important limitation is that total serum calcium was measured, whereas ionized calcium, the physiologically active fraction, was not assessed. Total calcium is influenced by serum albumin concentration, while blood pH affects calcium binding to albumin and therefore the proportion of ionized calcium. In CKD, hypoalbuminemia may occur in association with urinary albumin loss and other disease-related factors, potentially complicating the interpretation of total calcium concentrations. Because albumin-adjusted calcium was not calculated and ionized calcium was not measured, it remains uncertain whether the observed inverse association between total calcium and Glu-OC reflects differences in physiologically active calcium or, in part, variation in albumin concentration. Moreover, albumin-adjustment formulas may not reliably reflect ionized calcium in all patients with CKD. Direct measurement of ionized calcium, alongside serum albumin and blood pH, would provide a more physiologically informative assessment in future studies.

Additionally, the positive relationship between Glu-OC and P1NP (ρ = 0.382, *p* = 0.001; Table 3 and Figure 1) supports a link between Glu-OC and osteoblast-lineage cell activity. P1NP is a marker of type I collagen synthesis and bone formation [38], while osteocalcin is synthesized by osteoblasts [6]. The persistence of this association after adjustment (partial ρ = 0.297, *p* = 0.015) further supports the possibility that variations in Glu-OC in CKD are related, at least in part, to alterations in bone metabolism rather than being explained solely by kidney function.

### 3.5. FGF23, Calcium, and Glu-OC: A Potential Integrated Axis

Taken together, the findings suggest a functional relationship among kidney function, mineral homeostasis, FGF23, and osteocalcin metabolism. On the one hand, CKD progression is associated with a nonlinear pattern of FGF23 concentrations and changes in calcium–phosphate regulation. On the other hand, both FGF23 and calcium show independent associations with Glu-OC (Table 3 and Table 8).

**Figure 2:**
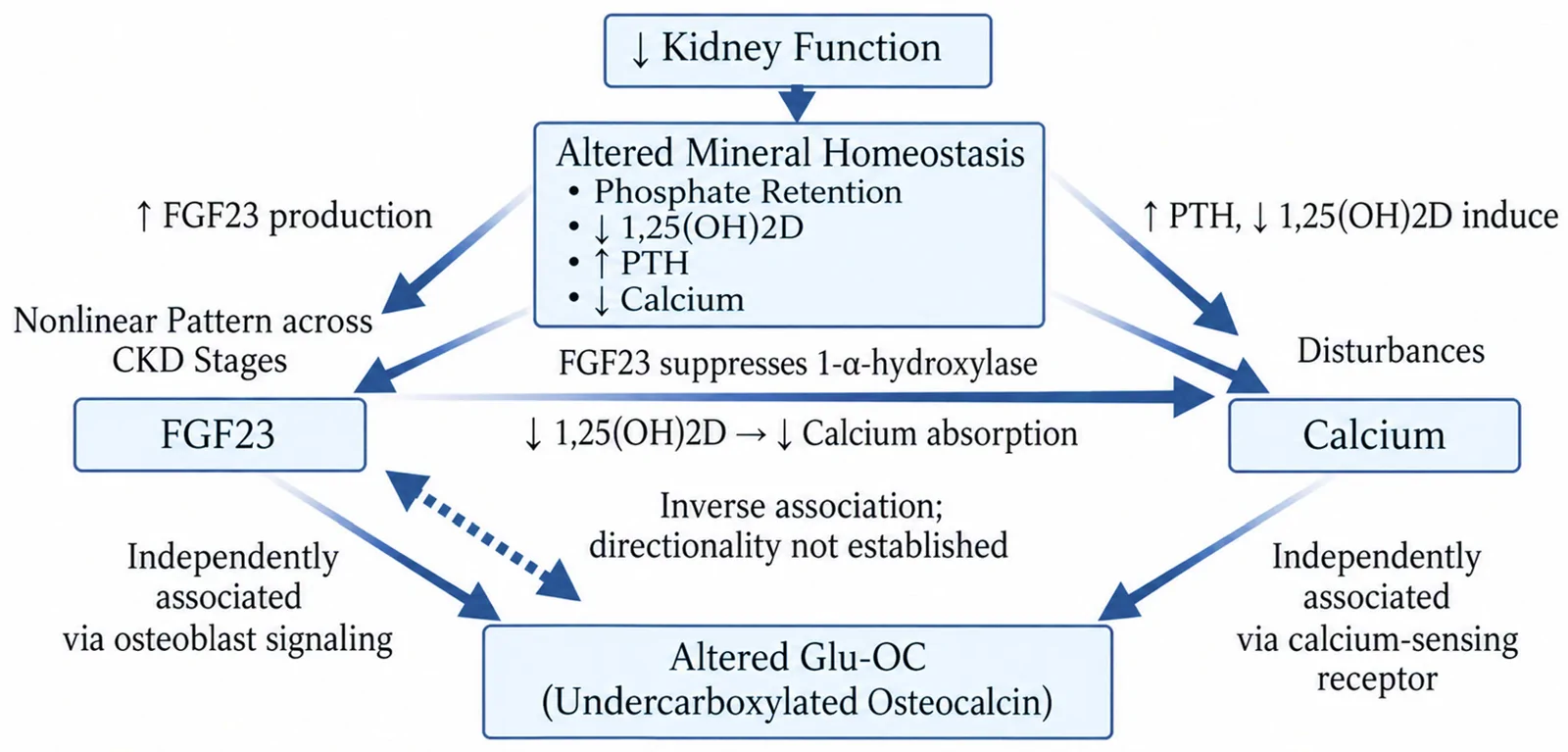
Proposed conceptual framework linking kidney function, mineral homeostasis, FGF23, calcium, and Glu-OC in CKD-MBD. The framework summarizes the observed associations and their potential biological interrelationships. It is hypothesis-generating and does not imply a unidirectional causal pathway or establish causality.

To integrate these findings within the broader context of CKD-MBD, a conceptual framework is proposed to illustrate the potential relationships among kidney function, mineral homeostasis, FGF23, calcium, and Glu-OC (Figure 2).

This model does not imply a unidirectional causal chain. Rather, it highlights the possibility that Glu-OC lies at the intersection of several interconnected regulatory mechanisms in CKD-MBD [34,36,37].

Of particular interest is that FGF23 and calcium are independently associated with Glu-OC, while their observed concentration patterns across CKD stages differ. This may help explain why the relationship between kidney function and Glu-OC is nonlinear and why Glu-OC concentrations do not change monotonically with advancing CKD stage (Table 1). Instead, Glu-OC may reflect the combined effects of renal, mineral, and bone regulatory mechanisms.

### 3.6. Role of α-Klotho

The absence of statistically significant differences in circulating α-Klotho concentrations across the study groups and the lack of significant correlations between α-Klotho and either FGF23 or Glu-OC (Table 1 and Table 3) do not exclude the involvement of Klotho-dependent signaling in the processes examined. Membrane-bound α-Klotho functions as a coreceptor for FGF23–FGFR signaling in target tissues, particularly in the kidney, whereas circulating soluble α-Klotho is generated predominantly through shedding of the extracellular domain of the membrane protein. Consequently, serum α-Klotho concentrations do not necessarily reflect tissue-specific membrane expression or local Klotho-dependent signaling activity [39,40].

The interpretation of circulating α-Klotho concentrations in CKD is further complicated by biological and analytical factors. Differences in measurement methods and assay performance may contribute to variability in reported concentrations and inconsistent associations with kidney function. Accordingly, circulating α-Klotho should not be regarded as a direct or comprehensive surrogate for tissue-level Klotho expression or signaling activity [40]. These considerations provide context for the present null findings but do not establish that the measurements obtained in this study were inaccurate.

Importantly, the absence of significant correlations between circulating α-Klotho and FGF23 or Glu-OC does not demonstrate that the FGF23–Glu-OC association operates independently of Klotho. Although both Klotho-dependent and potentially Klotho-independent actions of FGF23 have been discussed in the literature, the physiological significance and tissue specificity of Klotho-independent signaling remain incompletely resolved [39,41]. Therefore, the present findings cannot establish whether the observed association reflects Klotho-dependent signaling, Klotho-independent effects, shared regulatory influences, or a combination of these mechanisms.

The inverse association between FGF23 and Glu-OC remained statistically significant after simultaneous adjustment for eGFR and circulating α-Klotho (partial correlation coefficient = −0.481, *p* < 0.001; Table 4). This suggests that the association was not fully accounted for by these measured variables. Nevertheless, adjustment for circulating α-Klotho cannot establish independence from membrane-bound Klotho or from Klotho-dependent signaling within bone or other target tissues. As tissue expression, receptor activity, and downstream signaling pathways were not directly assessed, the contribution of Klotho-dependent and Klotho-independent mechanisms to the observed association remains undetermined.

Further studies combining well-characterized assays for circulating α-Klotho with functional investigations and, where feasible, tissue-level assessments of Klotho signaling are needed to clarify the role of Klotho in the relationship between FGF23 and Glu-OC in CKD.

### 3.7. Potential Biological and Clinical Significance

These findings have potential implications for understanding CKD-MBD, as they indicate that the association between FGF23 and Glu-OC persists after statistical adjustment for kidney function. This broadens the traditional view of FGF23 as primarily a phosphate-regulating hormone and places its relationship with bone cell function in a wider context [33].

Glu-OC is also of interest because of its proposed extraskeletal effects. Osteocalcin has been described as a potentially active endocrine mediator that may participate in the regulation of glucose and energy metabolism [12,42,43]. However, the present study did not include direct metabolic endpoints and therefore cannot establish whether changes in Glu-OC have functional implications for insulin sensitivity or glucose metabolism in CKD.

From a clinical perspective, the findings suggest that simultaneous assessment of FGF23 and Glu-OC may provide additional information about the interplay between kidney dysfunction and bone and mineral metabolism. However, this possibility requires evaluation in larger cohorts and longitudinal studies to determine whether these biomarkers provide additional clinical value beyond eGFR and standard CKD-MBD parameters.

The combined FGF23–Glu-OC profile may also warrant investigation as a means of characterizing biochemical subphenotypes of CKD-MBD. In particular, the FGF23/Glu-OC ratio could be explored as a candidate composite marker. However, neither this ratio nor its incremental value over the individual biomarkers and established CKD-MBD parameters was evaluated in the present study. Its reproducibility, discriminatory performance, and clinical relevance therefore require assessment in independent cohorts.

A second hypothesis is that interventions that modify FGF23 concentrations may be accompanied by changes in circulating Glu-OC. Whether such changes occur, and whether they reflect a direct biological relationship or indirect effects mediated by kidney function, calcium–phosphate homeostasis, PTH, vitamin D, or other regulatory pathways, remains unknown. Longitudinal studies and interventional investigations with serial measurements of FGF23, Glu-OC, and established CKD-MBD markers could help determine whether changes in these biomarkers are temporally associated and whether their combined assessment provides clinically meaningful information.

Given the cross-sectional design of the present study, these possibilities should be regarded as testable hypotheses rather than evidence of diagnostic utility, causal relationships, or therapeutic effects. Further independent and prospective studies are needed to determine whether these hypotheses have clinical relevance.

### 3.8. Strengths and Limitations

A major strength of this study is the simultaneous assessment of FGF23, Glu-OC, Gla-OC, P1NP, CTX1, ALP, PTH, calcium, phosphate, total 25(OH)D, and α-Klotho in a well-defined group of patients with non-dialysis CKD stages G1–G4. This enabled examination of the FGF23–Glu-OC relationship in the context of kidney function, mineral homeostasis, and bone turnover.

An additional strength is the use of partial correlation analyses and multivariable regression. Of particular relevance, the inverse association between FGF23 and Glu-OC remained statistically significant after adjustment for eGFR (Table 3), suggesting that the association was not fully explained by the measured level of kidney function.

The study also has several limitations. First, its cross-sectional design precludes establishing temporal sequence or causal relationships. Therefore, the findings should be interpreted as associations, and the proposed mechanisms as biological hypotheses.

Second, the sample size was relatively small, particularly for the stage-specific analyses. This likely limits statistical power and may contribute to the lack of statistical significance in G4.

Third, no direct assessments of bone histomorphometry, bone mineral density, or tissue expression of FGF23/Klotho were performed. Consequently, it is not possible to determine directly whether the observed serum associations reflect specific changes in bone tissue.

Fourth, the use of total serum calcium rather than ionized or albumin-adjusted calcium limits the mechanistic interpretation of the inverse relationship between calcium and Glu-OC.

Fifth, due to resource constraints, biochemical parameters were evaluated via single measurements rather than in duplicate. To minimize analytical error, all samples were analyzed simultaneously within a single run under strict quality control. Furthermore, while a few extreme values in advanced CKD stages could exert leverage in linear models, the stability of these biological associations was safeguarded by non-parametric Spearman correlations, rank-transformed partial correlations, and robust bootstrapping (10,000 resamples) with BCa 95% CIs, confirming that trends were not driven by individual leverage points or artifacts.

Finally, future studies should include larger independent cohorts, longitudinal follow-up, and direct measures of bone and mineral biology to determine whether the FGF23–Glu-OC association provides additional clinical information beyond established CKD-MBD parameters and whether it has prognostic relevance.

## 4. Materials and Methods

### 4.1. Study Population and Design

This cross-sectional study included 80 adults aged 33–84 years: 12 healthy controls and 68 patients with non-dialysis chronic kidney disease (CKD) stages G1–G4, classified according to the Kidney Disease: Improving Global Outcomes (KDIGO) criteria [44]. The estimated glomerular filtration rate (eGFR) was calculated using the 2021 CKD-EPI creatinine equation and expressed in mL/min/1.73 m^2^ [45]. The health status of the control participants was assessed based on medical history, clinical examination, and laboratory investigations, including serum creatinine measurement, eGFR assessment, and urinary protein testing. Control participants with clinical or laboratory findings suggestive of kidney disease were not included. Participants with acute kidney injury, CKD stage G5 or dialysis treatment, kidney transplantation, active liver disease, active inflammatory or infectious conditions, or clinically significant metabolic bone diseases were excluded.

### 4.2. Ethical Considerations

All participants provided written informed consent before enrollment. The study was conducted in accordance with the ethical principles for medical research outlined in the Declaration of Helsinki and was approved by the Research Ethics Committee of the Medical University–Pleven.

### 4.3. Blood Sample Collection and Serum Preparation

Venous blood samples were collected in the morning after an overnight fast. Samples were centrifuged at 2500 rpm for 10 min to obtain serum. Serum samples were processed and, when required, diluted according to the manufacturers’ instructions. All laboratory analyses were performed in a certified medical laboratory in accordance with standard operating procedures.

### 4.4. Routine Biochemical and Immunochemical Measurements

Serum creatinine (SCr), inorganic phosphate (Pi), and alkaline phosphatase (ALP) were measured using a Roche cobas c 311 clinical chemistry analyzer (Minato-ku, Tokyo, 105-6409 Japan). Parathyroid hormone (PTH) and total 25-hydroxyvitamin D [25(OH)D] were measured using a Roche cobas e 411 analyzer with electrochemiluminescence immunoassay (ECLIA) technology (Roche Diagnostics, Mannheim, Germany).

### 4.5. ELISA Measurements

Serum concentrations of undercarboxylated osteocalcin (ucOC; Glu-OC), carboxylated osteocalcin (cOC; Gla-OC), fibroblast growth factor 23 (FGF23), procollagen type I N-terminal propeptide (P1NP), C-terminal telopeptide of type I collagen (CTX-I), and α-Klotho were determined using commercially available enzyme-linked immunosorbent assay (ELISA) kits according to the manufacturers’ instructions.

Glu-OC was measured using the Human Undercarboxylated Osteocalcin (ucOC) ELISA Kit (MyBioSource, San Diego, CA, USA; Cat. No. MBS700581), whereas Gla-OC was measured using the Human Carboxylated Osteocalcin ELISA Kit (MyBioSource; Cat. No. MBS753090). FGF23, P1NP, and CTX-I were measured using the Human FGF23 ELISA Kit (Cat. No. E-EL-H1116), Human P1NP ELISA Kit (Cat. No. E-EL-H0185), and Human CTX-I ELISA Kit (Cat. No. E-EL-H0835), respectively (Elabscience Bionovation Inc., Houston, TX, USA). Serum α-Klotho was measured using the Human KL (Klotho) ELISA Kit (Elabscience Bionovation Inc.; Cat. No. E-EL-H5451).

All assays were performed in accordance with the manufacturers’ protocols and standard laboratory procedures.

### 4.6. Statistical Analysis

Statistical analyses were performed using IBM SPSS Statistics version 27.0 (IBM Corp., Armonk, NY, USA). Continuous variables were assessed for normality using the Shapiro–Wilk test and visual inspection of histograms and quantile–quantile (Q–Q) plots. Normally distributed continuous variables are presented as mean ± standard deviation (SD), whereas non-normally distributed variables are presented as median and interquartile range (IQR). Categorical variables are expressed as number and percentage.

Between-group comparisons were performed using one-way analysis of variance (ANOVA) for normally distributed continuous variables and the Kruskal–Wallis test for non-normally distributed variables, as appropriate. Categorical variables were compared using Pearson’s chi-square (χ^2^) test. Pairwise post-hoc comparisons following the Kruskal–Wallis test were performed using Dunn’s test with Bonferroni adjustment for multiple comparisons.

Associations between continuous variables were assessed using Spearman’s rank correlation coefficient (ρ). Partial correlation analyses were conducted using rank-transformed variables to assess associations between FGF23 or Glu-OC and the investigated biochemical and bone turnover parameters while adjusting for estimated glomerular filtration rate (eGFR). The association between FGF23 and Glu-OC was additionally examined using partial correlation analyses adjusted for eGFR alone, circulating α-Klotho alone, and both eGFR and α-Klotho.

Spearman correlation analyses between FGF23 and Glu-OC were performed separately in the control group and in each CKD stage group (G1–G4). To characterize the uncertainty of the group-specific correlation estimates, bootstrap standard errors and bias-corrected and accelerated (BCa) 95% confidence intervals (CIs) were calculated from 10,000 resamples. Post-hoc statistical power estimates for these group-specific Spearman correlations were calculated using an asymptotic normal approximation and a variance estimation approach for correlation coefficients. These estimates were interpreted cautiously because post-hoc power depends on the observed effect size and does not independently establish the reliability or absence of an association. The bootstrap confidence intervals were used to characterize uncertainty in the individual correlation estimates and were not interpreted as formal tests of differences between correlation coefficients across groups.

Two complementary multivariable linear regression models were fitted using backward elimination. Serum FGF23 concentration was specified as the dependent variable in the first model, and serum Glu-OC concentration was specified as the dependent variable in the second model. The initial models included sex, age, eGFR, total serum calcium, inorganic phosphate, total 25-hydroxyvitamin D, alkaline phosphatase (ALP), procollagen type I N-terminal propeptide (P1NP), C-terminal telopeptide of type I collagen (CTX-1), hypertension, diabetes mellitus, vitamin D supplementation, and vitamin K2 supplementation. Glu-OC was additionally included as a candidate predictor in the FGF23 model, whereas FGF23 was included as a candidate predictor in the Glu-OC model. Hypertension, diabetes mellitus, and vitamin D and vitamin K2 supplementation were included as potential confounding variables in the initial models. Calcium-containing preparations and phosphate binders were not included because none of the participants received these therapies. Binary categorical variables were entered as indicator variables. Backward elimination was used to derive the final models.

Regression results are reported as unstandardized coefficients (B), standard errors (SE), standardized coefficients (β), t-statistics, 95% CIs, and *p*-values. Overall model significance was evaluated using the F-test. Model fit was assessed using the coefficient of determination (R^2^), adjusted R^2^, and standard error of the estimate. Selected associations were illustrated using scatter plots with fitted simple linear regression lines and corresponding coefficients of determination (R^2^).

All statistical tests were two-sided, and *p* < 0.05 was considered statistically significant.

## 5. Conclusions

In this cross-sectional study of patients with non-dialysis CKD, serum FGF23 was independently and inversely associated with Glu-OC, and this association persisted after adjustment for eGFR. The finding was supported by complementary multivariable regression models. Glu-OC was also inversely associated with serum calcium and positively associated with P1NP, indicating relationships with mineral homeostasis and bone turnover.

Stage-specific analyses revealed significant inverse correlations between FGF23 and Glu-OC in CKD stages G1–G3, but not in stage G4 or the control group. These subgroup findings should be interpreted cautiously given the limited sample sizes and uncertainty of the group-specific estimates; differences in statistical significance do not necessarily indicate differences between correlation coefficients.

Overall, these results support an association between FGF23 and Glu-OC in non-dialysis CKD and provide a rationale for further investigation of a potential FGF23–Glu-OC axis in CKD-MBD. However, the cross-sectional design does not establish causality or clarify the underlying biological mechanisms. Larger longitudinal studies and mechanistic investigations are needed to determine the nature of this association and whether it provides clinically meaningful information beyond established markers of kidney function and mineral and bone metabolism.

## Figures and Tables

**Table 1:** Descriptive characteristics and biochemical parameters of the study groups.

Parameters	Controls (*n* = 12)	CKD G1 (*n* = 18)	CKD G2 (*n* = 16)	CKD G3 (*n* = 18)	CKD G4 (*n* = 16)	*p*-Value
Women, *n* (%)	8 (66.7%)	13 (72.2%)	6 (37.5%)	9 (50.0%)	6 (37.5%)	0.152
Age (years) ^1^	51.17 ± 10.49	56.39 ± 13.74	65.19 ± 9.07	67.39 ± 9.14	64.44 ± 13.23	<0.001 *
eGFR (mL/min/1.73 m^2^) ^2^	103.00 (93.00–117.00)	96.50 (91.00–111.75)	69.50 (63.00–77.75)	44.50 (34.75–48.50)	16.50 (15.00–20.75)	<0.001 *
SCr (µmol/L) ^2^	78.50 (71.00–87.00)	61.00 (55.25–74.00)	94.00 (86.50–103.50)	142.00 (109.00–159.50)	284.50 (250.25–432.00)	<0.001 *
Ca (mmol/L) ^1^	2.50 ± 0.09	2.38 ± 0.09	2.38 ± 0.08	2.37 ± 0.12	2.31 ± 0.19	0.004 *
Pi (mmol/L) ^2^	1.18 (1.16–1.26)	1.02 (0.97–1.18)	1.18 (1.08–1.32)	1.11 (1.03–1.27)	1.52 (1.23–1.69)	<0.001 *
ALP (U/L) ^2^	60.00 (44.00–74.00)	73.00 (67.75–95.00)	71.00 (55.50–85.75)	79.00 (69.75–100.75)	93.50 (67.25–121.75)	0.012 *
PTH (pg/mL) ^2^	26.30 (24.09– 29.80)	18.85 (11.34– 49.82)	25.95 (19–43.48)	42.28 (31.61– 54.18)	110.35 (32.19– 170.1)	<0.001 *
Total 25(OH)D (nmol/L) ^2^	53.55 (48.60–59.50)	60.7 (51.49–68.35)	50.12 (35.20–69.76)	44.1 (33.33–62.62)	35.85 (30.08–41.83)	0.001 *
Glu-OC (ng/mL) ^2^	0.59 (0.32–4.48)	2.93 (0.85–6.30)	3.15 (0.76–6.18)	5.22 (1.86–6.96)	4.01 (2.78–6.67)	0.033 *
Gla-OC (ng/mL) ^1^	3.51 ± 0.33	3.10 ± 0.33	3.56 ± 0.53	3.36 ± 0.39	3.35 ± 0.54	0.035 *
FGF23 (pg/mL) ^2^	182.87 (125.00–215.93)	83.40 (63.32–130.92)	184.80 (108.74–248.06)	178.67 (74.62–289.20)	208.65 (120.94–265.44)	0.023 *
α-Klotho (ng/mL) ^2^	21.77 (6.90–25.55)	20.95 (14.29–30.25)	19.80 (14.80–24.07)	23.68 (14.64–29.75)	25.95 (9.78–41.01)	0.722
P1NP (pg/mL) ^2^	2487 (1928–3784)	2666 (1649–3428)	2783 (2329–4865)	4187 (3171–6226)	5450 (4924–12,389)	<0.001 *
CTX-1 (ng/mL) ^2^	1.86 (1.34–2.70)	1.39 (1.16– 1.69)	1.15 (1.00–1.54)	1.95 (1.30–2.15)	1.63 (1.25–2.66)	0.033 *

Note: Data are presented as mean ± standard deviation (SD) ^1^ or median (interquartile range, IQR) ^2^, as appropriate. Between-group comparisons were performed using one-way analysis of variance (ANOVA) ^1^ or the Kruskal–Wallis test ^2^. Categorical variables were compared using Pearson’s chi-square test. * *p* < 0.05 was considered statistically significant. Abbreviations: CKD, chronic kidney disease; eGFR, estimated glomerular filtration rate; SCr, serum creatinine; Ca, calcium; Pi, inorganic phosphate; ALP, alkaline phosphatase; PTH, parathyroid hormone; 25(OH)D, 25-hydroxyvitamin D; Glu-OC, undercarboxylated osteocalcin; Gla-OC, carboxylated osteocalcin; FGF23, fibroblast growth factor 23; α-Klotho, alpha-Klotho; P1NP, procollagen type I N-terminal propeptide; CTX-1, C-terminal telopeptide of type I collagen.

**Table 2:** Dunn–Bonferroni post-hoc pairwise comparisons of FGF23 and Glu-OC across study groups.

Post-Hoc Pairwise Comparisons of FGF23					
Sample 1-Sample 2	Test Statistic	Std. Error	Std. Test Statistic	Unadjusted *p*-Value	Adjusted *p*-Value
G1-G2	−18.101	7.978	−2.269	0.023	0.233
G1-G3	−20.194	7.740	−2.609	0.009	0.091
G1-Controls	20.944	8.653	2.420	0.016	0.155
G1-G4	−23.194	7.978	−2.907	0.004	0.036 *
G2-G3	−2.094	7.978	−0.262	0.793	1.000
G2-Controls	2.844	8.867	0.321	0.748	1.000
G2-G4	−5.094	8.209	−0.620	0.535	1.000
G3-Controls	0.750	8.653	0.087	0.931	1.000
G3-G4	−3.000	7.978	−0.376	0.707	1.000
Controls-G4	−2.250	8.867	−0.254	0.800	1.000
**Post-hoc pairwise comparisons of Glu-OC**					
**Sample 1-Sample 2**	**Test statistic**	**Std. error**	**Std. test statistic**	**Unadjusted *p*-value**	**Adjusted *p*-value**
Controls-G1	−12.222	8.659	−1.411	0.158	1.000
Controls-G2	−13.479	8.873	−1.519	0.129	1.000
Controls-G3	−23.278	8.659	−2.688	0.007	0.072
Controls-G4	−24.917	8.873	−2.808	0.005	0.050
G1-G2	−1.257	7.984	−0.157	0.875	1.000
G1-G3	−11.056	7.745	−1.427	0.153	1.000
G1-G4	−12.694	7.984	−1.590	0.112	1.000
G2-G3	−9.799	7.984	−1.227	0.220	1.000
G2-G4	−11.438	8.215	−1.392	0.164	1.000
G3-G4	−1.639	7.984	−0.205	0.837	1.000

Abbreviations: FGF23, fibroblast growth factor 23; Glu-OC, undercarboxylated osteocalcin; G1–G4, study groups; Controls, control group; Sig., unadjusted *p*-value; Adj. Sig., Bonferroni-adjusted *p*-value. * *p* < 0.05 indicates statistical significance.

**Table 3:** Spearman correlations and eGFR-adjusted partial correlations between FGF23, Glu-OC, and markers of renal function, mineral metabolism, and bone turnover in patients with chronic kidney disease (CKD; *n* = 68).

Correlations of FGF23				
Variable	Spearman *ρ*	*p*-Value	Partial Correlation Coefficient (Rank-Transformed Variables, Adjusted for eGFR)	*p*-Value
eGFR	−0.346	0.004 *	–	–
SCr	0.299	0.013 *	–	–
Glu-OC	−0.344	0.004 *	−0.475	<0.001 *
P1NP	0.052	0.671	−0.284	0.020 *
PTH	0.186	0.130	−0.019	0.879
Pi	0.257	0.034 *	0.103	0.406
Ca	0.194	0.112	0.271	0.027 *
Total 25(OH)D	−0.149	0.226	0.002	0.987
α-Klotho	−0.074	0.550	−0.098	0.482
CTX-1	0.103	0.401	0.051	0.684
ALP	0.286	0.018 *	0.232	0.059
**Correlations of Glu-OC**				
eGFR	−0.252	0.038 *	–	–
SCr	0.272	0.025 *	–	–
FGF23	−0.344	0.004 *	−0.475	<0.001 *
P1NP	0.382	0.001 *	0.297	0.015 *
PTH	0.180	0.143	0.043	0.732
Pi	0.134	0.276	0.009	0.941
Ca	−0.405	<0.001 *	−0.382	0.001 *
Total 25(OH)D	−0.093	0.452	0.020	0.874
α-Klotho	−0.013	0.917	−0.027	0.828
CTX-1	−0.159	0.195	−0.210	0.088
ALP	−0.067	0.585	−0.128	0.304

Note: Spearman’s rank correlation coefficient (ρ) was used to assess unadjusted associations. Partial correlation coefficients were calculated using rank-transformed variables, with adjustment for eGFR. Dashes indicate that adjusted partial correlations were not calculated for eGFR and serum creatinine. Abbreviations: FGF23, fibroblast growth factor 23; Glu-OC, undercarboxylated osteocalcin; eGFR, estimated glomerular filtration rate; SCr, serum creatinine; P1NP, procollagen type I N-terminal propeptide; PTH, parathyroid hormone; Pi, inorganic phosphate; Ca, serum calcium; 25(OH)D, 25-hydroxyvitamin D; α-Klotho, alpha-Klotho; CTX-1, C-terminal telopeptide of type I collagen; ALP, alkaline phosphatase. * *p* < 0.05 was considered statistically significant.

**Table 4:** Partial correlations between FGF23 and Glu-OC under alternative adjustment models.

Adjustment Model	Control Variable(s)	Partial Correlation Coefficient (Rank-Transformed Variables)	*p*-Value
Model 1	eGFR	−0.475	<0.001 *
Model 2	eGFR + α-Klotho	−0.481	<0.001 *
Model 3	α-Klotho	−0.346	0.004 *

Note: Partial correlations were calculated using rank-transformed variables. Degrees of freedom (df) were 65 for Models 1 and 3 and 64 for Model 2. Abbreviations: eGFR, estimated glomerular filtration rate; FGF23, fibroblast growth factor 23; Glu-OC, undercarboxylated osteocalcin; α-Klotho, alpha-Klotho. * *p* < 0.05 indicates statistical significance.

**Table 5:** Spearman correlations and post hoc statistical power for the association between FGF23 and Glu-OC in controls and across CKD stages G1–G4.

Group	Variables	Spearman’s *ρ*	*p*-Value	Post-Hoc Power
Controls (*n* = 12)	FGF23–Glu-OC	−0.086	0.791	0.058
CKD G1 (*n* = 18)	FGF23–Glu-OC	−0.768	<0.001 *	0.933
CKD G2 (*n* = 16)	FGF23–Glu-OC	−0.511	0.043 *	0.481
CKD G3 (*n* = 18)	FGF23–Glu-OC	−0.541	0.021 *	0.591
CKD G4 (*n* = 16)	FGF23–Glu-OC	−0.366	0.163	0.268

Note: Spearman’s rank correlation coefficient (ρ) was used to assess the association between fibroblast growth factor 23 (FGF23) and undercarboxylated osteocalcin (Glu-OC). CKD stages were classified according to estimated glomerular filtration rate (eGFR): G1, ≥90; G2, 60–89; G3, 30–59; and G4, 15–29 mL/min/1.73 m^2^. * *p* < 0.05 indicates statistical significance. Abbreviations: CKD, chronic kidney disease; eGFR, estimated glomerular filtration rate; FGF23, fibroblast growth factor 23; Glu-OC, undercarboxylated osteocalcin.

**Table 6:** Bootstrap standard errors and BCa 95% confidence intervals for Spearman correlations between FGF23 and Glu-OC in controls and across CKD stages G1–G4.

Group	Bootstrap SE	BCa 95% CI
Controls (*n* = 12)	0.389	−0.740 to 0.696
CKD G1 (*n* = 18)	0.107	−0.958 to −0.342
CKD G2 (*n* = 16)	0.193	−0.807 to −0.002
CKD G3 (*n* = 18)	0.197	−0.821 to −0.010
CKD G4 (*n* = 16)	0.242	−0.740 to 0.182

Note: Bootstrap estimates were obtained from 10,000 resamples. The BCa confidence intervals characterize uncertainty in the group-specific correlation estimates and do not constitute formal tests of differences between correlation coefficients across groups. Abbreviations: BCa, bias-corrected and accelerated; CI, confidence interval; SE, standard error.

**Table 7:** Multivariable linear regression analysis of factors associated with serum FGF23 concentrations in patients with chronic kidney disease (CKD; *n* = 68): initial and final models.

Model		Unstandardized Coefficients		Standardized Coefficients	*t*	Sig.	95.0% Confidence Interval for *B*	
		*B*	Std. Error	*β*			Lower Bound	Upper Bound
1	(Constant)	192.314	824.260	–	0.233	0.816	−1460.942	1845.569
	Gender	−40.102	75.106	−0.074	−0.534	0.596	−190.745	110.541
	Years	0.490	3.750	0.022	0.131	0.897	−7.032	8.011
	eGFR (mL/min/1.73 m^2^)	−2.238	1.572	−0.269	−1.424	0.160	−5.391	0.914
	Ca (mmol/L)	54.545	332.548	0.026	0.164	0.870	−612.463	721.552
	Pi (mmol/L)	−81.370	149.480	−0.092	−0.544	0.588	−381.188	218.448
	Total 25(OH)D (nmol/L)	1.120	1.047	0.142	1.070	0.290	−0.980	3.219
	ALP (U/L)	1.865	0.702	0.430	2.656	0.010 *	0.456	3.274
	Glu-OC (ng/mL)	−17.231	8.905	−0.259	−1.935	0.058	−35.093	0.630
	P1NP (pg/mL)	−0.002	0.016	−0.029	−0.151	0.880	−0.034	0.029
	CTX1 (ng/mL)	−0.015	14.080	0.000	−0.001	0.999	−28.256	28.227
	Hypertension	−66.673	117.059	−0.087	−0.570	0.571	−301.463	168.117
	Diabetes mellitus	44.544	78.553	0.076	0.567	0.573	−113.013	202.101
	Vit. D (suppl.)	92.453	81.461	0.171	1.135	0.262	−70.937	255.844
	Vit. K2 (suppl.)	−66.468	83.271	−0.110	−0.798	0.428	−233.489	100.554
12	(Constant)	252.640	98.243	–	2.572	0.012 *	56.377	448.903
	eGFR	−1.884	0.957	−0.226	−1.970	0.053	−3.795	0.027
	ALP (U/L)	1.600	0.481	0.369	3.329	0.001 *	0.640	2.560
	Glu-OC (ng/mL)	−17.132	7.396	−0.258	−2.316	0.024 *	−31.907	−2.357

Note: Serum FGF23 concentration was the dependent variable. Model 1 included all candidate predictors, whereas Model 12 represents the final model obtained by backward elimination. Final model statistics were as follows: R = 0.518, R^2^ = 0.269, adjusted R^2^ = 0.235, and standard error of the estimate = 238.52 pg/mL. Abbreviations: B, unstandardized regression coefficient; SE, standard error; β, standardized regression coefficient; t, t-statistic; CI, confidence interval; eGFR, estimated glomerular filtration rate; Ca, total serum calcium; Pi, inorganic phosphate; 25(OH)D, 25-hydroxyvitamin D; ALP, alkaline phosphatase; Glu-OC, undercarboxylated osteocalcin; P1NP, procollagen type I N-terminal propeptide; CTX-1, C-terminal telopeptide of type I collagen; suppl., supplementation. * *p* < 0.05 was considered statistically significant.

**Table 8:** Multivariable linear regression analysis of factors associated with serum Glu-OC concentrations in patients with chronic kidney disease (CKD; *n* = 68): initial and final models.

Model		Unstandardized Coefficients		Standardized Coefficients	*t*	Sig.	95.0% Confidence Interval for *B*	
		*B*	Std. Error	*β*			Lower Bound	Upper Bound
1	(Constant)	29.983	11.583	–	2.588	0.012 *	6.750	53.216
	Gender	0.170	1.122	0.021	0.151	0.880	−2.081	2.421
	Years	0.114	0.054	0.342	2.129	0.038 *	0.007	0.222
	eGFR (mL/min/1.73 m^2^)	−0.024	0.024	−0.192	−1.018	0.313	−0.071	0.023
	Ca (mmol/L)	−12.182	4.668	−0.385	−2.610	0.012 *	−21.544	−2.820
	Pi (mmol/L)	−0.923	2.231	−0.069	−0.414	0.681	−5.397	3.552
	Total 25(OH)D (nmol/L)	−0.007	0.016	−0.057	−0.431	0.668	−0.038	0.025
	ALP (U/L)	−0.007	0.011	−0.108	−0.636	0.527	−0.029	0.015
	FGF 23 (pg/mL)	−0.004	0.002	−0.254	−1.935	0.058	−0.008	0.000
	P1NP (pg/mL)	0.000	0.000	0.136	0.722	0.473	0.000	0.001
	CTX1 (ng/mL)	−0.013	0.210	−0.009	−0.062	0.951	−0.434	0.408
	Hypertension	2.204	1.724	0.192	1.278	0.207	−1.254	5.661
	Diabetes mellitus	−1.184	1.163	−0.134	−1.018	0.313	−3.517	1.149
	Vit. D (suppl.)	1.129	1.219	0.139	0.926	0.359	−1.316	3.574
	Vit. K2 (suppl.)	−1.927	1.220	−0.212	−1.579	0.120	−4.375	0.521
12	(Constant)	25.122	8.378	–	2.998	0.004 *	8.385	41.860
	Years	0.118	0.038	0.353	3.105	0.003 *	0.042	0.194
	Ca (mmol/L)	−11.565	3.643	−0.366	−3.175	0.002 *	−18.842	−4.287
	FGF 23 (pg/mL)	−0.004	0.002	−0.266	−2.392	0.020 *	−0.007	−0.001

Note: Serum Glu-OC concentration was the dependent variable. Model 1 included all candidate predictors, whereas Model 12 represents the final model obtained by backward elimination. Final model statistics were as follows: R = 0.481, R^2^ = 0.231, adjusted R^2^ = 0.195, and standard error of the estimate = 3.68 ng/mL. Abbreviations: B, unstandardized regression coefficient; SE, standard error; β, standardized regression coefficient; t, t-statistic; CI, confidence interval; eGFR, estimated glomerular filtration rate; Ca, total serum calcium; Pi, inorganic phosphate; 25(OH)D, 25-hydroxyvitamin D; ALP, alkaline phosphatase; Glu-OC, undercarboxylated osteocalcin; FGF23, fibroblast growth factor 23; P1NP, procollagen type I N-terminal propeptide; CTX-1, C-terminal telopeptide of type I collagen; suppl., supplementation. * *p* < 0.05 was considered statistically significant.

## Data Availability

The original contributions presented in this study are included in the article. Further inquiries can be directed to the corresponding author.

## Abbreviations

The following abbreviations are used in this manuscript:

ALP	Alkaline Phosphatase
CKD	Chronic Kidney Disease
CKD-EPI	Chronic Kidney Disease Epidemiology Collaboration
CKD-MBD	Chronic Kidney Disease–Mineral and Bone Disorder
Ca	Calcium
CTX1	Cross-Linked C-Telopeptide of Type I Collagen
ECLIA	Electrochemiluminescence Immunoassay
eGFR	Estimated Glomerular Filtration Rate
FGF23	Fibroblast Growth Factor 23
Glu-OC	Undercarboxylated Osteocalcin
Gla-OC	Carboxylated Osteocalcin
KDIGO	Kidney Disease: Improving Global Outcomes
P1NP	Procollagen Type I N-Propeptide
P3NP	Procollagen Type III N-Propeptide
Pi	Inorganic Phosphate
PTH	Parathyroid Hormone
SCr	Serum Creatinine
Total 25(OH)D	Total 25-Hydroxyvitamin D
